# Association of Genetic polymorphism of *PPARγ-2, ACE, MTHFR, FABP-2 and FTO* genes in risk prediction of type 2 diabetes mellitus

**DOI:** 10.1186/1423-0127-20-80

**Published:** 2013-10-25

**Authors:** Shania Abbas, Syed Tasleem Raza, Faisal Ahmed, Absar Ahmad, Saliha Rizvi, Farzana Mahdi

**Affiliations:** 1 Molecular Biology Lab, Department of Biochemistry, Era’s Lucknow Medical College and Hospital, Lucknow 226003, India

**Keywords:** Genome sequencing, Single nucleotide polymorphism, Genetic polymorphism, Peroxisome proliferator-activated receptor gamma, Angiotensin converting enzyme, Methylene tetrahydrofolate reductase, Fatty acid binding protein-2, Fat mass and obesity associated gene, Type 2 diabetes mellitus

## Abstract

Type 2 diabetes mellitus (T2DM) is a non-autoimmune, complex, heterogeneous and polygenic metabolic disease condition characterized by persistent elevated blood glucose levels (hyperglycemia). India as said to be the diabetic capital of the world is likely to experience the largest increase in T2DM and a greater number of diabetic individuals in the world by the year 2030. Identification of specific genetic variations in a particular ethnic group has a critical role in understanding the risk of developing T2DM in a much efficient way in future. These genetic variations include numerous types of polymorphisms among which single nucleotide polymorphisms (SNPs) is the most frequent. SNPs are basically located within the regulatory elements of several gene sequences. There are scores of genes interacting with various environmental factors affecting various pathways and sometimes even the whole signalling network that cause diseases like T2DM. This review discusses the biomarkers for early risk prediction of T2DM. Such predictions could be used in order to understand the pathogenesis of T2DM and to better diagnostics, treatment, and eventually prevention.

## Review

### Overview of type 2 diabetes mellitus

T2DM is a chronic metabolic disorder with a rapidly increasing prevalence highlighting the importance of continued research and the need for novel methods to both prevent and treat this pandemic disease. In case of India, the disease burden is estimated to be 87 million around 2030 [1]. The negative impacts of T2DM are considerable: as a lifelong disease, which increases morbidity and mortality, ultimately decreasing the quality of life [2]. If diabetes is not efficiently controlled, then the patient has a significantly higher risk of developing complications such as, hypoglycemia, ketoacidosis, and non-ketotic hyperosmolar coma. Apart from these, long-standing complications could be cardiovascular disease, chronic kidney failure, retinal damage, nerve damage, poor healing of wounds, gangrene on the feet leading to amputation, and erectile dysfunction etc. The recent global epidemic of T2DM almost certainly indicates the importance of environmental triggers such as sedentary lifestyle and dietary changes over last several decades. Nevertheless T2DM is amongst those complex diseases for which genetic contribution is well accepted. Identification of genetic components of T2DM is the most important area of diabetes research because elucidation of the diabetes genes (alleles) will influence all efforts toward a mechanistic understanding of the disease, its complications, cure, treatment and prevention [3]. Basically, many genes perform key regulatory functions in the development of T2DM, which is a polygenic disorder with multiple genes located on different chromosomes contributing to its susceptibility. The analysis of genetic factors associated with T2DM is further complicated by the fact that a variety of environmental factors interact with these genes to produce the disorder. Thus, identification and characterization of the gene variants among a particular ethnic group that play a significant role in T2DM, is one of the most important areas of diabetes research because it will influence all efforts toward a mechanistic understanding of the disease complications, treatment, cure and prevention.

### Candidate genes for type 2 diabetes mellitus

Numerous reports have been published on the genetics of T2DM with most recent ones showcasing the effect of SNP’s in various genes corresponding to risk prediction of T2DM such as, gene variants of Peroxisome Proliferator-Activated Receptor Gamma (PPAR-γ) [4-6], Angiotensin Converting Enzyme (ACE) [7-9], Methylene Tetrahydrofolate Reductase (MTHFR) [10-14], Fatty Acid Binding Protein-2 (FABP2) [15-19] and Fat Mass and Obesity associated gene (FTO) [20,21]. In this review, we will focus on candidate genes (PPAR-γ, ACE, MTHFR, FABP2 and FTO) in which the genetic variants have been well established to be functional and shown in more than one study for their association with T2DM, in various ethnic groups. Findings from Previously Conducted Meta-Analyses of different Gene Variant in T2DM are shown in Table 1.

**Table 1:** **A comparative study of *PPARγ2, ACE, MTHFR, FABP2 *** **and *FTO *** **genes polymorphism with T2DM in various ethnic groups**

***PPARG2*** **Ethinicity**	**Reference**	**OR**	**P value**	**95%CI**	**Significant (Y/N)**
Japanese	[22]	4.35	<0.05		Y
USA	[23]	1.24	<0.05	0.99–1.57	Y
UK	[24]	0.86	<0.05	0.81, 0.90	Y
Caucasians	[25]	0.81	<0.05	0.72. 0.91	Y
Europeans	[26]	0.81	<0.05	0.75, 0.88	Y
North Indian	[27]	0.65	<0.05	0.42-0.99	Y
USA	[21]	0.12	<0.05	0.03–0.52	Y
French population	[28]	1.37	<0.05		Y
***ACE***					
**Ethinicity**	**Reference**	**OR**	**P value**	**95%CI**	**Significant (Y/N)**
North Indian	[29]		<0.05		Y
Indian	[30]	8.826	<0.05	1.012,76.96	Y
Malaysian	[9]		>0.05		N
Taiwanese	[31]		<0.05		Y
Iranian	[10]	3.122	<0.05	1.12-8.64	Y
Japanese	[32]	1.49	<0.05	1.01-2.21	Y
UK	[7]	1.55	<0.05	0.89-2.60	Y
Turkish	[33]		<0.05		Y
Australian	[34]	1.16	>0.05	0.94-1.43	N
Caucasians	[35]		>0.05		N
***MTHFR***					
**Ethinicity**	**Reference**	**OR**	**P value**	**95%CI**	**Significant (Y/N)**
Turkish	[11]	3.76	<0.05	1.28-11.00	Y
Brazilian	[13]		>0.05		N
Chinese	[14]	4.04	<0.05	1.95-8.34	Y
North Indian	[27]	0.54	<0.05	0.29-0.98	Y
***FABP2***					
**Ethinicity**	**Reference**	**OR**	**P value**	**95%CI**	**Significant (Y/N)**
North Indian	[29]		>0.05		N
USA	[19]		<0.05		Y
***FTO***					
**Ethinicity**	**Reference**	**OR**	**P value**	**95%CI**	**Significant (Y/N)**
North Indian	[21]	1.46	<0.05	1.11–1.93	Y
South Asian Indians	[36]		<0.05		Y
South African	[37]		>0.05		N
Spanish	[38]	0.97	>0.05	0.85-1.16	N
Scotland	[39]		<0.05		Y

### Peroxisome Proliferator-Activated Receptor Gamma (*PPAR-γ*) gene

PPARs (isoforms α, δ, and γ) are ligand-activated transcription factors that heterodimerise with retinoid X receptor (RXR). It has been shown that agonists of PPAR possess antidiabetogenic, anti-inflammatory, and antioxidant effects. PPAR-γ gene, encoding the nuclear receptor PPAR-γ, was the first gene reproducibly associated with T2DM [36]. PPAR-γ gene is located on chromosome 3p25 encodes a nuclear transcription factor involved in the expression of hundreds of genes. PPAR-γ gene contains 9 exons, spans more than 100 kilobases, because of alternative mRNA splicing results in the production of 2 protein isoforms: PPARγ-1 and PPARγ-2 [22]. PPARs constitute a distinct sub-family of the nuclear receptors that are activated by naturally occurring fatty acids [40]. The association between the substitution of alanine for proline at codon 12 of PPAR- γ and the risk for T2DM has been widely studied since Yen CJ, first reported this polymorphism [41]. Within a unique domain of PPARγ-2 gene that enhances ligand independent activation, a common Pro12Ala polymorphism has been identified [5]. This polymorphism has been reported to be associated with obesity. Using a family based design to control for population stratification, it was reported that Ala allele of this polymorphism was associated with a decreased risk of T2DM [36]. A meta-analysis conducted by Ludovico found that the alanine polymorphism conferred significantly greater protection against T2DM among Asians than Caucasians [23], contradictory result have been reported within the same study elsewhere where Ala12 variant was associated with a reduced risk for the development of diabetes [5,26,42]. Soon, it became apparent that most negative studies had been underpowered and after combining the data from all published studies in a meta-analysis it became evident that Pro12Ala variant was associated with T2DM [5,28,43]. PPAR-γ plays a critical role in glucose homeostasis and serves as the molecular target for a class of insulin-sensitizing drugs called thiazolidinediones (TZDs). TZDs is PPARγ2 ligands and widely used for treatment of T2DM [24], they had very minimal activity toward PPAR-α or PPAR-β. Although PPAR- γ levels are 10–30 times higher in fat than in muscle or liver, this receptor is expressed in these latter tissues. Effects on insulin action in other tissues would then occur as a consequence of alterations in signalling molecules produced by fat, such as free fatty acids, TNF-α , leptin, or others (Perspectives in Diabetes PPAR- γ: Adipogenic Regulator and TZDs Receptor) [44]. PPAR- γ activation controls one or more genes that regulate systemic insulin sensitivity like TNF-α and leptin.

### Angiotensin Converting Enzyme (ACE) gene

Genetic studies have revealed that the genes of renin angiotensin are highly polymorphic, raising the possibility in addition to environmental factors. The genetic make-up of Renin Angiotensin System (RAS) affects the status of RAS in the individuals. The emerging picture of ACE function is that it is more than just a key enzyme that catalyses cleavage of angiotensin I to the potent vasoconstrictor peptide angiotensin II [45]. ACE also hydrolyzes the inactive angiotensin (1–9) peptide into the vasodilator metabolite angiotensin (1–7) [46], and it is additionally thought to inactivate the vasodilator peptides bradykinin and kallidin [46]. Among the various SNP’s associated with RAS, one of the examples is insertion (I)/deletion (D) polymorphism of ACE gene, which consists of 26 exons and span 21 kb on chromosome 17. The polymorphism exists within intron 16, consisting the presence or absence of 287 bp fragment [47]. The II (Insertion-Insertion) genotype is reported to be protective against development and progression of diabetic and non-diabetic nephropathy to chronic kidney disease [48]. Clinically, serum ACE level is useful for the evaluation of disease activity and follow-up in T2DM [31,49-52]. All previous studies in non-diabetic and diabetic nephropathies have demonstrated that the deletion polymorphism of ACE gene, particularly the homozygote DD, is a risk factor for an accelerated loss of kidney function [53]. Studies of ACE gene with T2DM have shown contradictory results, where some have shown association of ACE gene with T2DM [7,9,31-34] while others have shown no such association [8,35]. Indian studies, reported a strong association of ACE gene polymorphisms with T2DM in Northern India [29]. Vishwanathan and Bhavani established positive association of ACE polymorphism with T2DM in south India [54,55]; while Prasad and Ajay Kumar reported no any relation between ACE gene and T2DM among North Indian population [56,57].

### Methylene Tetrahydrofolate reductase (MTHFR) gene

Methylenetetrahydrofolate reductase (MTHFR) has a major impact on regulating the folic acid pathway; it catalyzes the irreversible conversion of 5, 10‒methylenetretrahydrofolate, which is the methyl donor (for the conversion of dUMP to dTMP), into 5‒methyltetrahydrofolate. Genetic and environmental (e.g., dietary) factors play a key role in affecting the homocysteine levels [58]. One of the most common genetic defects of homocysteine metabolism is a mutation in MTHFR gene. The gene encoding MTHFR is located at 1p36.3 [59]. Studies have shown an association between homocysteine levels and diabetic complications such as macroangiopathy, retinopathy and nephropathy in T1DM, whereas no such association was seen among T2DM subjects [60]. Numerous polymorphisms in MTHFR gene have been reported. Frosst et al. described a C677T substitution at of MTHFR gene that converts an alanine to a valine residue [61]. In a few studies, no association was found between hyperhomocysteinemia, MTHFR gene C677T polymorphism, metabolic 6 syndromes and T2DM [62]. Nevertheless, in another study a significant correlation between these polymorphisms and individuals with normal weight and increased risks of developing metabolic syndrome (normal weight obese syndrome) was observed [63]. A high concentration of homocysteine was seen in patients with diabetes mellitus [64]. The mutant homozygous genotype for MTHFR C677T showed high risk of diabetic retinopathy among the individuals with T2DM [65]. Likewise, Ksaizek and co-workers have also found that MTHFR C677T mutation in MTHFR gene predisposes T2DM patients to the development of diabetic retinopathy [66]. These common polymorphisms are also associated with hyper homocysteinemia that has been reported to be an increased risk factor for neural tube defects, diabetes and cardiovascular diseases.

### Fatty acid binding protein 2 (FABP2) gene

FABP2 plays a key role in the absorption and intracellular transport of dietary long chain fatty acids. Since, glucose and fatty acid metabolism are inter-related phenomenons, FABP2 soon became an important candidate gene for T2DM. In the search for T2DM loci in Pima Indians, Prochazka and co-workers found linkage between insulin resistance and a region on chromosome 4q near the FABP2 locus [67]. This finding is supported by a positive linkage between post challenge insulin levels and FABP2 in Mexican–Americans [68]. Molecular scanning of FABP2 identified a missense mutation (Ala54Thr) responsible for insulin resistance [69]. Carriers of the Thr54 allele in FABP2 have a twofold greater affinity for the absorption for the long-chain fatty acids than those with the Ala54-containing FABP2 [70]. Genotypic/Phenotypic studies involving FABP2 have focussed on the Ala–›Thr (G–›A) substitution in exon-2 which is responsible for increased binding affinity, transport of long chain fatty acids and more efficient secretion of triglycerides in cells expressing Thr-encoding allele [69,71]. In a study of Canadian Oji-Cree Indians, the Thr encoding allele was associated with increased BMI, percent body fat, and plasma triglycerides [72]. FABP2 Ala54Thr variant has been associated with an increased fasting insulin concentration, increased rate of lipid oxidation, reduced insulin-stimulated glucose uptake and increased concentrations of fasting and postprandial triglyceride-rich lipoprotein [19,70,71,73-75]. It has been suggested that the Ala54Thr polymorphism might associate with the risk for atherosclerosis because it causes a compositional change in LDL particles [76], an altered postprandial lipemia [70]. Previous studies have found contradictory associations between FABP2 genotypes and the occurrence of T2DM, obesity or decreased insulin sensitivity [17,70,71,77-79]. Contradictory to it, several studies have reported the association between the Ala54Thr polymorphism of FABP2 with insulin resistance and T2DM [17,19,69,80-82]. In contrast, studies in other Japanese [83,84], Caucasians [85,86], and African-American [87] cohorts have not found an association of T2DM, insulin levels, or obesity with the Thr54 variant.

### Fat Mass and Obesity associated gene (FTO)

Fat mass and obesity associated (FTO) gene was found in a genome-wide association (GWA) study for T2DM and showed to predispose individuals to diabetes through an effect on Body mass Index (BMI). The FTO gene, which is located on chromosome 16q12.2 consists nine exons and emerged 450 million years ago [88]. FTO is primarily expressed in the hypothalamus and encodes a 2-oxoglutarate-dependent nucleic acid demethylase. Sequence analysis suggested that FTO has homology with the AlkB family of DNA repair enzymes. Subsequent in vitro biochemical studies revealed FTO to be a member of the Fe (II) and 2-oxoglutarate (2OG) dependent oxygenase superfamily [89]. In metazoans these enzymes are involved in diverse processes including oxygen sensing, DNA repair, fatty acid metabolism and post-translational modifications [90]. A number of SNPs in tight linkage disequilibrium with rs9939609, and residing in the first intron of the FTO gene, had been associated with obesity in large populations of adults and children. Recently, part of a genome-wide association study found that SNPs of the FTO were strongly associated with obesity and T2DM [91,92]. FTO gene encodes for a protein 2-oxoglutarate dependent nucleic acid demethylase involved in fatty acid metabolism, DNA repair and post- translational modifications [93]. It may also play important roles in the management of energy homeostasis [88,94], nucleic acid demethylation, and regulation of body fat masses by lipolysis [95]. The hypothalamic expression of FTO suggests a potential role in the control of food intake and whole body metabolism wherein physical activity and food intake is unchanged but metabolic rate is increased. The association of FTO variants with T2DM and BMI has been independently identified in a number of white European populations [96] but the findings are somewhat inconsistent in Asians, which may be the result of varying study designs, inadequate sample sizes or ethnic differences [97-99]. A recent study in north Indian Sikhs demonstrated a strong association of FTO variants with type 2 diabetes, which did not seem to be mediated through BMI [21]. This raises empirical probability that the association of FTO variants with BMI and T2DM might be different in Asian populations. FTO gene confers risk for T2DM in Europeans, with each A allele increasing BMI by approximately 0.4 kg/m2 [100]. However, results have been variable for replication in other ethnic populations such as Hispanics [101], Asians, Oceanics [102] and Blacks [103].

## Conclusion

Multiple genes are involved in pathogenesis of T2DM, each contributing a small amount to the overall risk making T2DM, a truly complex disorder. Our understanding of genetics of diseases gives a better prospective of biochemical and molecular mechanism of disease on the whole. The data could help to identify at-risk patients in early stages and may provide opportunities for early prevention. Our better understanding of such phenomena will throw new light on how common variants can alter disease susceptibility and it is necessary to understand the physiologic importance of the genetic associations those are uncovered. The utility of genetic approaches will depend on a holistic understanding of the interactions among the genes and also between genes and the environment. Combining these genetic variations with new developments in the fields of bioinformatics, genomics, and proteomics will lead to a greater understanding of the pathogenesis of T2DM and may present new information on diagnostics, treatment and eventual prevention of the disease. Additionally, inclusion of genetic studies in design and analysis of drug trials could lead to development of genetic biomarkers that predict treatment response. Prediction can be made on the basis of biomarkers in connection to the course of disease; treatment-response and possibilities of side-effects will be vastly appreciated. As a result of wide range of investigations over the last few years, a few biomarkers have been introduced into clinical practices. This requires a personalized medicine approach. This genetic information may also form the basis for development of new drug therapies such as individually specific or targeted pharmacotherapy. Thus, an understanding of common variants and of the genetic/non-genetic factors with which they interact can improve public health by focussing on genetic individuality in the diagnosis and treatment of disease.

## Abbreviations

T2DM: Type 2 diabetes mellitus; ACE: Angiotensin converting enzyme; RAAS: Renin–angiotensin–aldosterone system; PPARγ: Peroxisome proliferator-activated receptor gamma; MTHFR: Methylene tetrahydrofolate reductase; FTO: Fat mass and obesity associated gene, type 2 diabetes mellitus; FABP: Fatty acid-binding proteins; SNPs: Single nucleotide polymorphisms; Pro: Proline; Ala: Alanine; Thr: Threonine; LDL: Low-density lipoprotein; GWA: Genome wide association; BMI: Body mass index.

## Competing interests

The authors declare that they have no competing interests.

## Authors’ contributions

SA and ST have done overall search and compilation of data. AA and SR help in the literature search and preparation of table. FA helps in the correction of grammatical and typological mistake. FM has done overall supervision. All authors read and approved the final manuscript.
